# Elevated nuclear localization of glycolytic enzyme TPI1 promotes lung adenocarcinoma and enhances chemoresistance

**DOI:** 10.1038/s41419-022-04655-6

**Published:** 2022-03-04

**Authors:** Peng Liu, Si-Jia Sun, Ying-Jie Ai, Xu Feng, Yi-Min Zheng, Yun Gao, Jin-Ye Zhang, Lei Zhang, Yi-Ping Sun, Yue Xiong, Miao Lin, Hai-Xin Yuan

**Affiliations:** 1 The Fifth People’s Hospital of Shanghai and the Molecular and Cell Biology Research Lab of the Institutes of Biomedical Sciences, Fudan University, Shanghai, China; 2 Department of Gastroenterology and Hepatology, Zhongshan Hospital, Fudan University, Shanghai, China; 3 Department of Liver Surgery, Zhongshan Hospital, Fudan University, Shanghai, China; 4 Cullgen Inc., 12671 High Bluff Drive, San Diego, CA 92130 USA; 5 Department of Thoracic Surgery, Zhongshan Hospital, Fudan University, Shanghai, China; 6 Center for Novel Target and Therapeutic Intervention, Institute of Life Sciences, Chongqing Medical University, Chongqing, 400016 China

**Keywords:** Non-small-cell lung cancer, Cell biology

## Abstract

Increased glycolysis is a hallmark of tumor, which can provide tumor cells with energy and building blocks to promote cell proliferation. Recent studies have shown that not only the expression of glycolytic genes but also their subcellular localization undergoes a variety of changes to promote development of different types of tumors. In this study, we performed a comprehensive analysis of glycolysis and gluconeogenesis genes based on data from TCGA to identify those with significant tumor-promoting potential across 14 types of tumors. This analysis not only confirms genes that are known to be involved in tumorigenesis, but also reveals a significant correlation of triosephosphate isomerase 1 (TPI1) with poor prognosis, especially in lung adenocarcinoma (LUAD). TPI1 is a glycolytic enzyme that interconverts dihydroxyacetone phosphate (DHAP) to glyceraldehyde 3-phosphate (GAP). We confirm the upregulation of TPI1 expression in clinical LUAD samples and an inverse correlation with the overall patient survival. Knocking down of *TPI1* in lung cancer cells significantly reduced cell migration, colony formation, and xenograft tumor growth. Surprisingly, we found that the oncogenic function of TPI1 depends on its translocation to cell nucleus rather than its catalytic activity. Significant accumulation of TPI1 in cell nucleus was observed in LUAD tumor tissues compared with the cytoplasm localization in adjacent normal tissues. Moreover, nuclear translocation of TPI1 is induced by extracellular stress (such as chemotherapy agents and peroxide), which facilitates the chemoresistance of cancer cells. Our study uncovers a novel function of the glycolytic enzyme TPI1 in the LUAD.

## Introduction

Lung cancer is one of the most common tumors and the leading cause of cancer-related death in the world, with 2.1 million people diagnosed and 1.76 million deaths annually [1]. Small cell lung cancer (SCLC) and non-SCLC (NSCLC) are the main types of lung cancer, accounting for 15% and 85%, respectively [2]. Lung adenocarcinoma (LUAD) is the most prevalent subtype of NSCLC, accounting for approximately 50% of all lung cancer cases [1]. LUAD patients usually have a poor prognosis, and often exhibit local progression or metastasis at diagnosis. Five-year survival is only 18% [3, 4]. The discovery of driver mutations and advancement in new sequencing technology allow the creation of personalized targeted treatment. Instead of empiric use of cytotoxic therapies, patients are tested for oncogenic drivers and receive effective and better-tolerated regimens that are targeted to specific molecular subtypes in LUAD [5–8]. For instance, epidermal growth factor receptor (EGFR) mutation-positive patients benefit greatly from EGFR tyrosine kinase inhibitors (EGFR TKIs), having a response rate as high as 80%, and around 10–14 months of progression-free survival (PFS) [9]. However, most patients develop drug resistance within about 1 year of EGFR TKIs treatment [10]. Therefore, identification of new molecular targets has high significance for new LUAD therapy.

Metabolic reprogramming, especially the altered glycolysis and gluconeogenesis pathway, is a common cancer hallmark [11–13]. Increased glycolysis is frequently observed in cancer cells and allows them to produce energy with lactic acid production even in the presence of oxygen, known as the “Warburg effect” [14, 15]. Glycolytic phenotype confers a proliferative growth advantage for invasive cancers, by generating ATP for energy supply, providing small molecules to meet requirements of macromolecular synthesis [16]. Three major types of regulations of glycolytic enzymes contribute to increased glycolysis: upregulated expression, enhanced enzyme activity, and altered subcellular localization. The increased expressions or activities of glycolytic enzymes provide more building blocks for cancer cells. The alteration of subcellular localization of glycolytic enzymes may promote cancer development through metabolic or non-metabolic functions [17, 18]. For instance, nucleus localized PKM2 could phosphorylate histones and transcription factors to promote the expression of oncogenes [19, 20]. Furthermore, it was recently reported that chemotherapeutic signals induce PFKFB3 acetylation and retain it in the cytosol, which promotes glycolysis and improve cell resistance to chemotherapy [21]. Thus, targeting the glycolytic pathway may preferentially injure the malignant cells and is likely to have broad therapeutic applications.

Triosephosphate isomerase 1 (TPI1) is a glycolytic enzyme that converts dihydroxyacetone phosphate (DHAP) to glyceraldehyde 3-phosphate (GAP). Mutations within the TPI coding region lead to a recessive disease known as TPI Deficiency, which is characterized by hemolytic anemia, neurologic dysfunction and often early death [22]. The mutation E104D shows reduced activity and is the most frequent in patients [23]. Upregulation of TPI1 has been reported in gastric cancer and pancreatic cancer. TPI1 could also promote the metastasis of gastric cancer cell lines [24]. Despite these reports, the knowledge or TPI1 in cancer is limited. The specific role of TPI1 in lung cancer development is unclear.

In this study, we observed that elevated TPI1 is an unfavorable prognostic factor for overall survival (OS) of LUAD patients. We further demonstrated that TPI1 is important for cell migration, colony formation and xenograft tumor growth of LUAD cells. Interestingly, we found the tumor promoting function of TPI1 is independent of its enzymatic catalytic activity, but dependent on its translocation to cell nucleus. This TPI1 nuclear translocation is induced by stress conditions and contributes to cell resistance to chemotherapy. Our study reveals a novel function of TPI1 in promoting LUAD.

## Results

### TPI1 is upregulated in lung adenocarcinoma

**Fig. 1:**
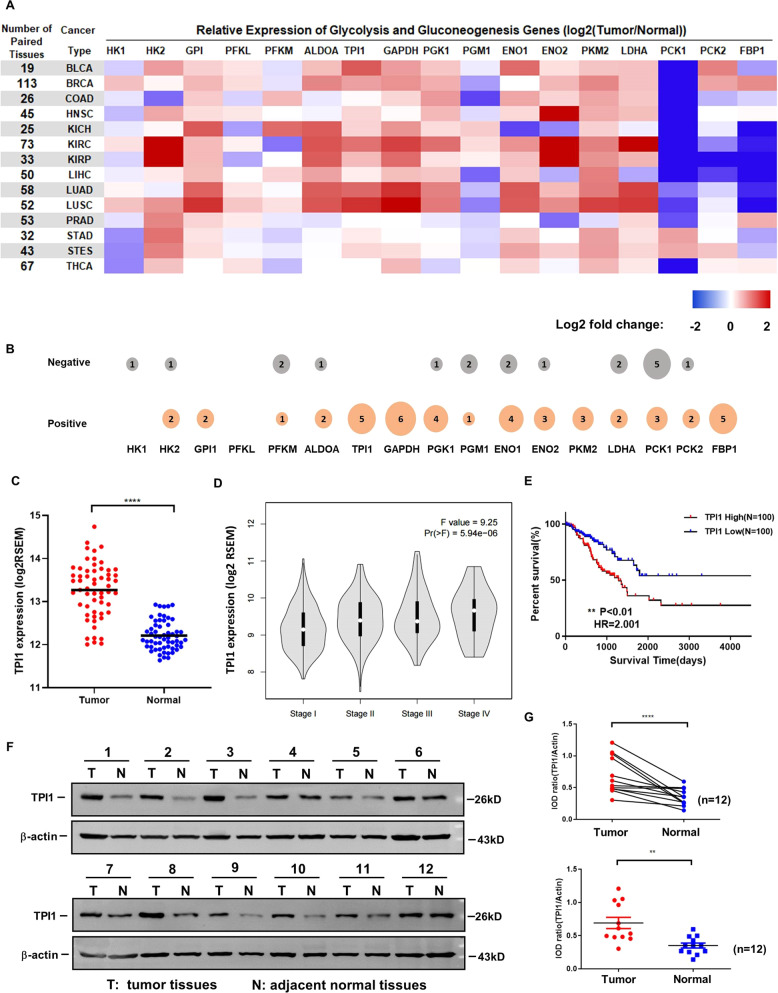
TPI1 is upregulated in lung adenocarcinoma. **A** Gene expression of key metabolic enzymes in glycolysis and gluconeogenesis pathway in 14 types of tumors from TCGA. Data were collected from Firehose Database (https://gdac.broadinstitute.org/). Log2((median of gene expression in tumor)/(median of gene expression in normal tissues)) was used to indicate the fold change of genes between tumor and normal. **B** The correlation between expression of metabolic enzymes and overall survival of tumor patients from TCGA. For each gene, the overall survival analysis in 14 type of cancer was conducted with R using the “ggsurvplot” package. The Orange represents that changes of gene expression result in shorter survival time while gray represents longer survival time. The size of bubble is correlated with the number of tumors conforming to the rule. **C** mRNA level of TPI1 in paired LUAD tumors (*n* = 58) and normal tissues (*n* = 58) from TCGA. Asterisks denote statistical significance with Student’s *t*-test. *p < 0.05. **D** GEPIA provides violin plots based on pathological stages in the ‘Expression DIY’ tab. **E** The relationship between TPI1 expression and survival time in LUAD using the Kaplan–Meier method with log-rank statistics. Data were from TCGA. **F** TPI1 protein expression in LUAD tumor and adjacent normal tissue. TPI1 was significantly upregulated in clinical LUAD samples compared to adjacent normal tissues. **G** Quantification of protein expression results in (**F**). Asterisks denote statistical significance with Student’s *t*-test. **p < 0.01, ****p < 0.0001.

To identify the key metabolic enzymes in glycolysis and gluconeogenesis pathway that are important for tumorigenesis, we performed a comprehensive expression analysis of these genes across 14 types of tumors with TCGA database. GPI1, TPI1, GAPDH, PGK1, and PKM2 were consistently upregulated in almost all tumor types, while PCK1, PCK2, and FBP1 were downregulated in tumors (Fig. 1A). To investigate the functional implication of the altered expression of these glycolytic enzymes in cancer, we did a combined analysis of gene expression data and overall survival data (Fig. 1B). Gene alteration correlated with a worse overall survival (OS) was marked with positive, gene alteration correlated with a better OS was marked with negative. We found that the expression of TPI1, GAPDH, PGK1, and FBP1 associates with worse survival. Previous work had illustrated the function of GAPDH, PGK1, and FBP1 in tumorigenesis [25–27], but the role of TPI1 has been rarely studied. Notably, TPI1 expression was significantly upregulated in LUAD tissues compared to normal tissues (Fig. 1C) and was correlated with advanced clinical grade and stage, as well as poorer survival (Fig. 1D, E). To confirm the result, we examined TPI1 expression in 12 pairs of clinical LUAD tumor and adjacent normal tissue samples, and found a significant increase of TPI1 protein level in LUAD tumors (Fig. 1F, G). The clinical information of enrolled patients was provided in Supplementary Table 1. All these results indicate TPI1 is upregulated in LUAD and may serve as a prognostic factor for LUAD patients.

### TPI1 is required for colony formation and migration of lung cancer cells independent of its catalytic activity

**Fig. 2:**
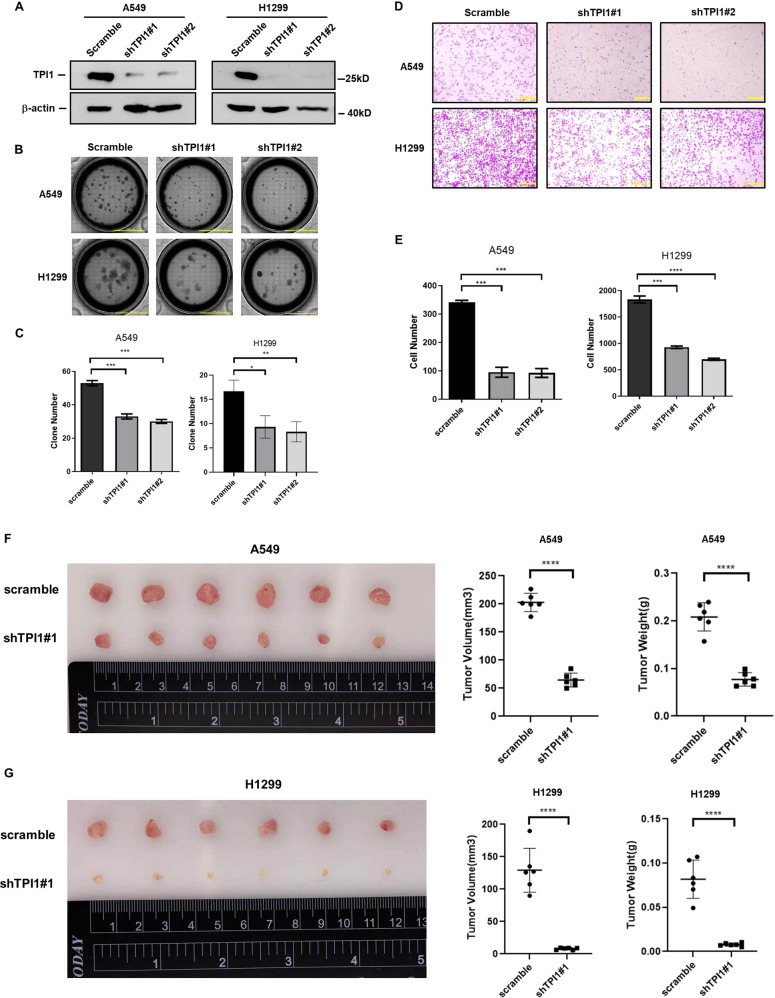
TPI1 is required for colony formation and migration ability of lung cancer cells. **A** Western blot validating knocking down of *TPI1* in A549 and H1299 cells. **B**, **C** Knocking down of *TPI1* reduced colony formation ability of A549 and H1299 cells. Bar graphs show the quantitative analysis data. Data were shown as mean ± SEM from experiments performed in triplicate. Asterisks denote statistical significance with one-way ANOVA. *p < 0.05; **p < 0.01; ***p < 0.001 for the indicated comparison. **D**, **E** Knocking down of *TPI1* reduced cell migration ability of A549 and H1299 cells. Bar graphs show the quantitative analysis data. Data were shown as mean ± SEM from experiments performed in triplicate. Asterisks denote statistical significance with one-way ANOVA. ***p < 0.001; ****p < 0.0001 for the indicated comparison. **F**, **G** Knocking down of *TPI1* reduced xenograft tumor growth of both A549 cells (**F**) and H1299 cells (**G**). Quantification of volume and weight of xenograft tumors were shown. Values are mean tumor volume or weight ± SD of six mice within each group. Asterisks denote statistical significance with Student’s *t*-test. *p < 0.05.

To explore the role of TPI1 in LUAD progression, we knocked-down TPI1 in A549 and H1299 cell lines with two different shRNAs (Fig. 2A). Colony-formation assay demonstrated that knocking down of *TPI1* in A549 cells and H1299 cells reduced the number of colonies compared to the Scramble control (Fig. 2B, C). The migratory capacity of A549 and H1299 cells was also impaired by TPI1 deficiency as determined by transwell assay (Fig. 2D, E). To verify the role of TPI1 in promoting tumorigenesis in vivo, xenograft tumor growth assay was conducted by injecting nude mice subcutaneously with control and *TPI1* knockdown A549 and H1299 cells. In accordance with the in vitro observation, *TPI1* knocking-down cells formed significantly smaller xenograft tumors than control cells, suggesting an oncogenic advantage of high TPI1 expression (Fig. 2F, G).

**Fig. 3:**
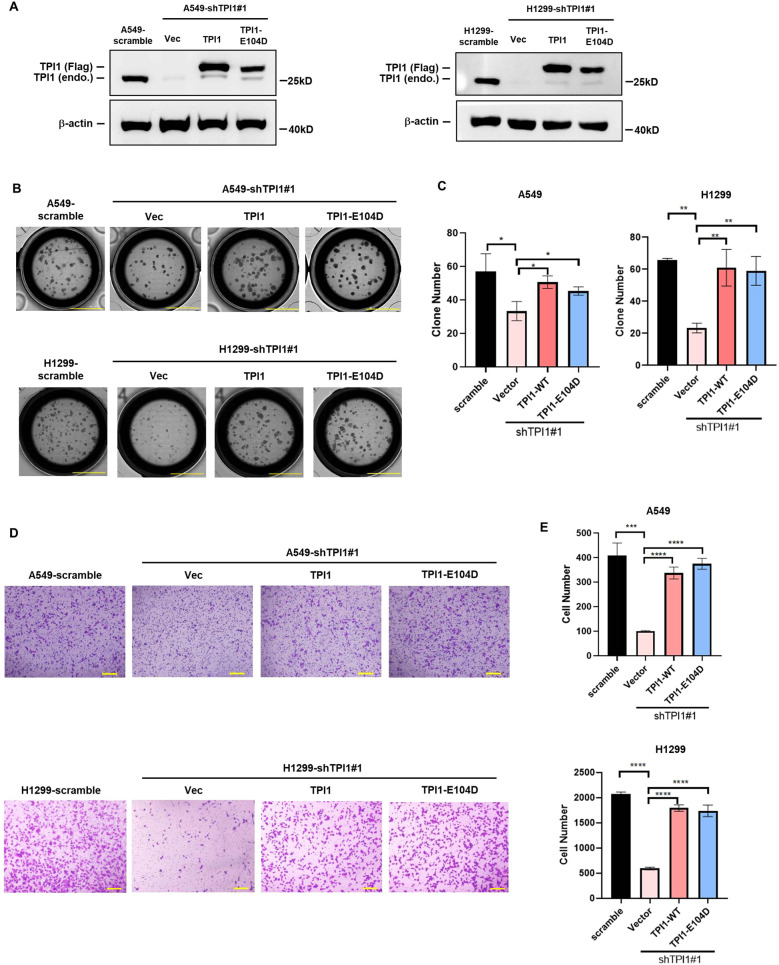
The oncogenic function of TPI1 is independent of its enzymatic activity. **A** Western blot validating ectopic expression of wide-type and E104D mutant TPI1 in A549 and H1299 cells. **B** Both wild-type and E104D mutant of TPI1 recovered colony formation ability of A549 and H1299 cells. **C** Bar graphs show the quantitative analysis data of colony formation assay in (**B**). Data were shown as mean ± SEM from experiments performed in triplicate. Asterisks denote statistical significance with one-way ANOVA. *p < 0.05 for the indicated comparison. **D** Both wild-type and E104D mutant of TPI1 recovered migration ability of A549 and H1299 cells. **E** Bar graphs show the quantitative analysis data of transwell assay in (**D**). Data were shown as mean ± SEM from experiments performed in triplicate. Asterisks denote statistical significance with one-way ANOVA. ***p < 0.001; ****p < 0.0001 for the indicated comparison.

To find out whether the oncogenic function of TPI1 depends on its catalytic activity, we ectopically expressed wide-type TPI1 and its E104D mutant, which impairs its stability and activity as a metabolic enzyme, in *TPI1* knockdown cells (Fig. 3A). Unexpectedly, re-expression of either wild-type or E104D mutant of TPI1 could recover the ability of colony formation (Fig. 3B, C) and migration (Fig. 3D, E) even though the E104D mutant was expressed at a lower level than the wide-type TPI1, indicating that the function of TPI1 in promoting the oncogenic capability of A549 and H1299 cells is independent of its enzymatic activity. In support of this, we found that knocking down of *TPI1* did not affect cell glycolysis rate under full-glucose culture condition as measured by the ECAR assay (Fig. S1).

### TPI1 accumulates in cell nucleus in LUAD tumor samples

**Fig. 4:**
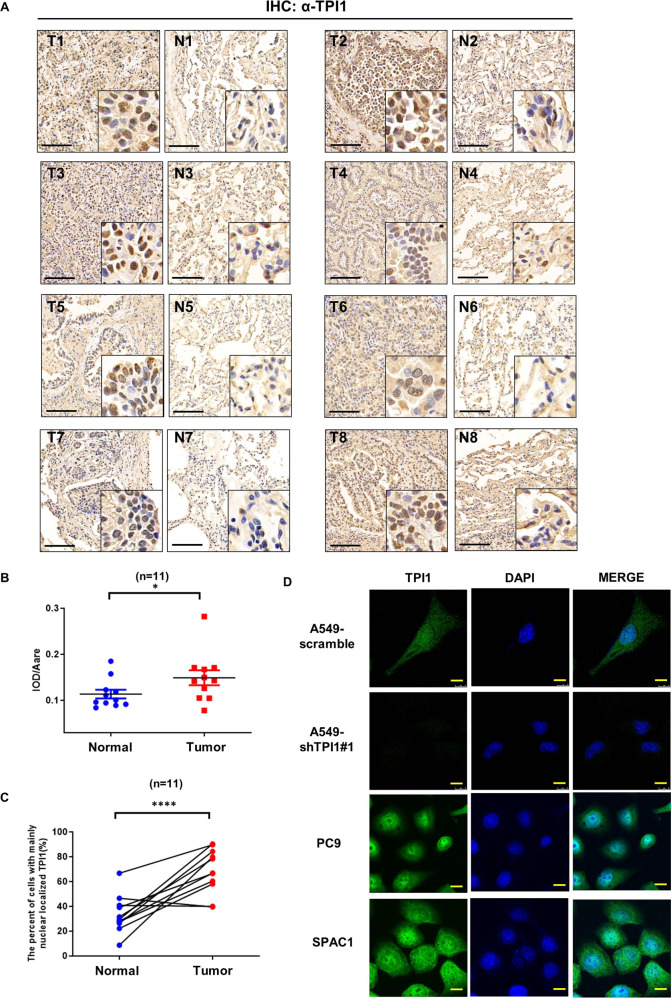
TPI1 is predominantly localized in cell nucleus in LUAD samples. **A** Representative IHC staining of TPI1 in paired LUAD and adjacent normal tissues. Scale Bar: 50 μm. **B** Quantification of TPI1 expression levels in IHC staining. The mean ratios of integrated optical density (IOD) to area (IOD/pixel) were used for analysis. Data are the mean ± SD from LUAD and adjacent normal tissues. Asterisks denote statistical significance with Student’s *t*-test. *p < 0.05. **C** Paired comparisons (Wilcoxon signed-rank test) of the percentage of cells with predominant nuclear localization of TPI1 revealed by IHC staining. Each line indicates the pair of tumor and adjacent normal tissue. ****p < 0.0001. **D** Localization of TPI1 in LUAD cell lines (A549, PC9, and SPAC1) revealed by Immunofluorescence staining. Notably, TPI1 signal was not detected in A549 cells with *TPI1* knockdown, demonstrating the specificity of the antibody. Scale Bar: 10 μm.

Next, we investigated the clinical relevance of TPI1 by immunohistochemistry (IHC) staining of TPI1 in LUAD samples. TPI1 exhibited significantly higher expression in tumor tissues than in adjacent normal tissues (Fig. 4A, B), which is consistent with the western blot result of LUAD tissues (Fig. 1F). Strikingly, we also observed predominant nuclear staining of TPI1 in tumor tissues, compared to the predominant cytoplasmic staining in normal tissues (Fig. 4A, C). This observation raised an interesting possibility that both upregulation and nuclear translocation are important for TPI1 to promote LUAD. Translocation of glycolytic enzymes to nucleus has been observed. However, to our knowledge, this is the first discovery that TPI1 undergoes nuclear translocation in LUAD. To confirm the above observation, we examined TPI1 localization in different LUAD cell lines by immunofluorescence. Consistently, TPI1 could be detected in both cytoplasm and nucleus of cells (Fig. 4D), as observed in tissue samples.

### Nuclear translocation of TPI1 promotes tumor cell migration and colony formation

**Fig. 5:**
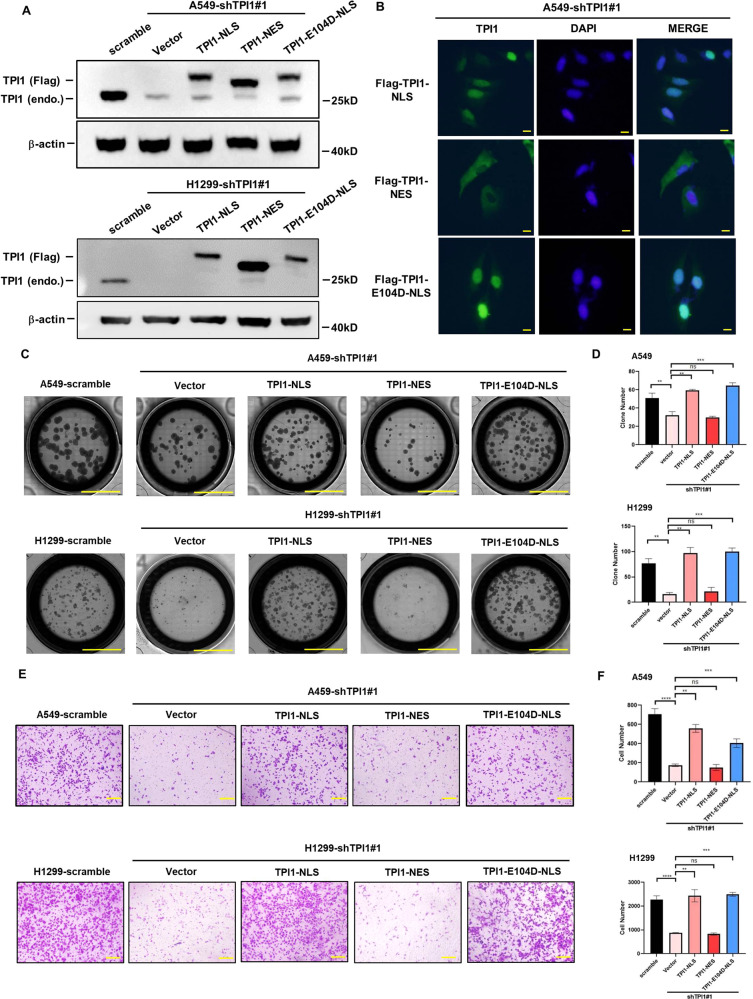
Nuclear translocation of TPI1 promotes tumor cell migration and colony formation. **A** Validation of ectopic expression of TPI1-NLS, TPI1-NES, and TPI1-E104D-NLS in A549 and H1299 cells. **B** Validation of localization of TPI1-NLS, TPI1-NES, and TPI1-E104D-NLS in A549 cells by immunofluorescence staining. Scale Bar: 50 μm. **C** TPI1 localized in nucleus rather than cytoplasm recovered colony formation ability of A549 and H1299 cells independent of enzymatic activity. **D** Bar graphs show the quantitative analysis data of colony formation assay in (**C**). Asterisks denote statistical significance with one-way ANOVA. **p < 0.01; ***p < 0.001 for the indicated comparison. **E** TPI1 localized in nucleus rather than cytoplasm recovered migration ability of A549 and H1299 cells independent of enzymatic activity. **F** Bar graphs show the quantitative analysis data of transwell assay in (**E**). Asterisks denote statistical significance with one-way ANOVA. **p < 0.01; ***p < 0.001; ****p < 0.0001 for the indicated comparison.

The nuclear localization of TPI1 in LUAD samples prompted us to investigate whether TPI1 translocation is important in promoting tumorigenesis. TPI1 protein sequence lacks typical nucleus-localization signal (NLS) or nuclear export signal (NES). To study the function TPI1 subcellular localization, we ectopically expressed TPI1 fused with an NLS or NES in *TPI1* knockdown cells (Fig. 5A). It is notable that both proteins were expressed at a level close to the endogenous level to mimic a physiological condition. Localization of TPI1-NLS in the nucleus and TPI1-NES in the cytoplasm was confirmed by immunofluorescence staining (Fig. 5B, S2). Interestingly, reconstitution with TPI1-NLS, rather than TPI1-NES, restored the colony formation (Fig. 5C, D) and migration capacity (Fig. 5E, F) of *TPI1* knockdown A549 and H1299 cells. Furthermore, we also expressed TPI1-NLS with E104D mutation and found that loss of enzyme activity did not influence its ability to restore the tumorigenic capacity of *TPI1* knockdown cells (Fig. 5A–F). These results suggest that the role of TPI1 in promoting LUAD development relies largely on its translocation to nucleus rather than its enzyme activity.

### TPI1 nuclear translocation is induced by oxidative stress and chemotherapy drugs, confers resistance to chemotherapy

**Fig. 6:**
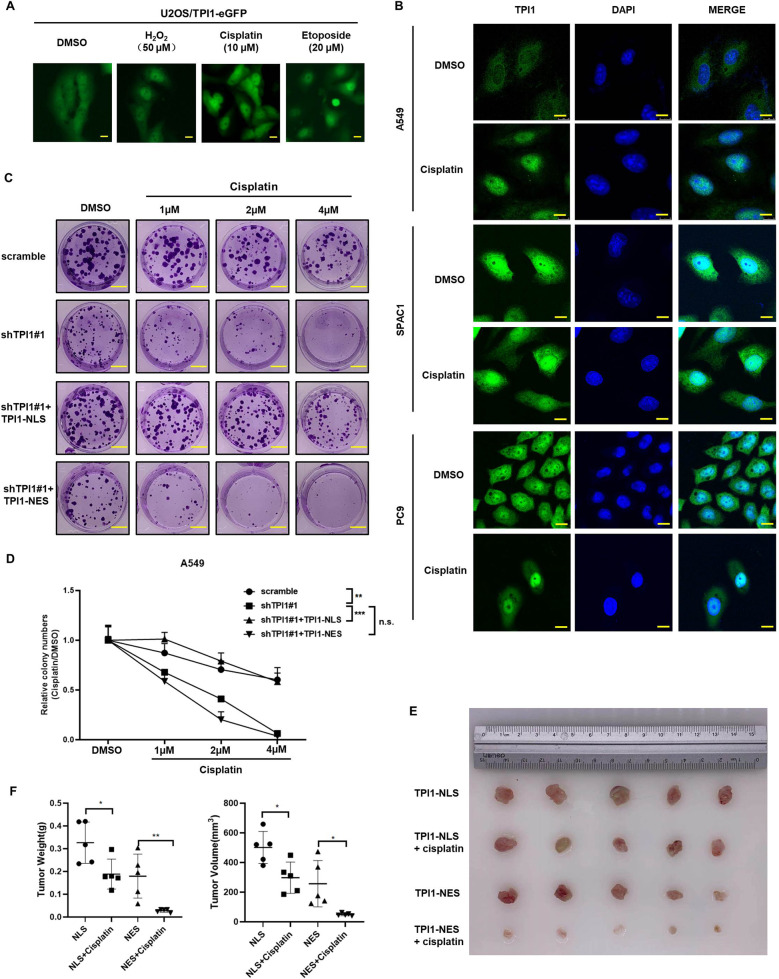
TPI1 nuclear translocation is induced by oxidative stress and chemotherapy drugs, confers resistance to chemotherapy. **A** Translocation of TPI1-GFP upon treatment with H_2_O_2_, cisplatin, or etoposide in U2OS cells. **B** Translocation of endogenous TPI1 in response to cisplatin or etoposide treatment in A549, SPAC1, and PC9 cells. **C** Colony formation upon cisplatin treatment in Scramble, *TPI1* knockdown, TPI1-NLS or TPI1-NES expressing cells. **D** Quantification of colony formation results in (**C**). Asterisks denote statistical significance with one-way ANOVA. **p < 0.01; ***p < 0.001 for the indicated comparison. **E** Xenograft tumors from TPI1-NES A549 cells are more resistant to cisplatin treatment than those from TPI1-NLS cells. **F** Quantification of weight and volume of xenograft tumors. Values are mean tumor weight/volume ± SD of five mice within each group. Asterisks denote statistical significance with one-way ANOVA. *p < 0.05; **p < 0.01.

Translocation of metabolic enzymes is often induced by change of physiological conditions. To investigate how TPI1 translocation is regulated, we determined TPI1 localization in response to various treatments, including peroxide, growth factors, serum starvation, glucose starvation and chemotherapeutic drugs. Notably, mild oxidative stress (H_2_O_2_) and chemotherapy drugs (cisplatin and etoposide) could promote TPI1 nucleus translocation in U2OS cells (Fig. 6A). Similar result was confirmed in multiple LUAD cell lines including A549, SPAC1, and PC9 (Fig. 6B). These results suggest that TPI1 subcellular localization may respond to cellular stress.

Based on the above findings, we hypothesized that TPI1 can act as a stress response protein and translocate into nucleus to promote cell survival. Since chemotherapy could induce TPI1 translocation, we tested whether this is associated with cell chemoresistance. Colony formation assay was performed with cisplatin treatment under different concentrations. The result showed that *TPI1* knockdown significantly increased cell sensitivity to cisplatin treatment in A549 cells (Fig. 6C, D) and H1299 cells (Fig. S3A, S3B). More strikingly, cells reconstituted with TPI1-NLS, but not TPI1-NES, rescued cells from ultra-sensitivity to cisplatin caused by *TPI1* knocking down, indicating that TPI1 nuclear translocation is important for cell protection under stress (Figs. 6C, 6D, S3A and S3B).

To gain more insight into the role of TPI1 translocation in tumor chemoresistance, we performed xenograft tumor growth assay by injecting nude mice subcutaneously with TPI1-NLS and TPI1-NES cells. After 7 days, each group was divided into two subgroups and treated with either DMSO or cisplatin through intraperitoneal injection. Cells with TPI1-NLS formed bigger xenograft tumors than TPI1-NES cells, which is consistent with the function of TPI1-NLS in promoting colony formation (Fig. 5). After cisplatin treatment, tumors formed by TPI1-NLS cells showed modest decrease of 44% in weight (0.326 ± 0.018 to 0.189 ± 0.059). In comparison, tumors formed by TPI1-NES cells was reduced by about 5 folds (0.180 ± 0.086 to 0.028 ± 0.006) upon cisplatin treatment (Fig. 6E, F). Similar change was also observed for tumor volume (Fig. 6E, F). The results strongly support that nuclear-localization of TPI1 induced by stress conditions promotes tumor cell survival and increases cell resistance to chemotherapy.

## Discussion

Metabolic enzymes participate in metabolic reprogramming in cancer cells to serve cellular metabolic needs through their canonical functions, but some metabolic enzymes also play non-canonical or non-metabolic roles, which are referred to “moon-lighting” functions in tumors [28, 29]. These noncanonical functions of metabolic enzymes are involved in various cellular activities, such as gene expression, cell proliferation, apoptosis and tumor microenvironment remodeling, and therefore contribute to tumorigenesis [30, 31].

In this article, we systematically screened metabolic enzymes in glycolysis and gluconeogenesis pathway which are essential for tumor development and found that TPI1 is upregulated in a variety of tumors and closely related to patients’ prognosis. TPI1 catalyzes the isomerization of DHAP and G3P, two products from hydrolysis of F-1,6-BP by aldolase but only G3P is the intermediate of glycolysis. TPI1 deficiency hinders the generation of G3P from DHAP, thus may partially impair glycolysis under limited glucose supply. However, under sufficient glucose condition, loss of TPI1 only blocked half flux from glucose and may not have much effect on glycolysis, as we observed with ECAR assay (Fig. S1A). In fact, this is supported by our observation that TPI1 prompts lung cancer cells proliferation, migration, and tumorigenesis independent of its enzymatic activity. To the best of our knowledge, this is the first finding that TPI1 exhibits tumor-promoting effect through non-metabolic functions.

In this report, we found that TPI1 is not only elevated, but also accumulated in nucleus LUAD samples (Fig. 4). Our data suggest surprising model that TPI1’s function in lung tumorigenesis is associated with nuclear translocation. In support of this, TPI1 with a NLS, but not NES, significantly promotes migration and colony formation ability of lung cancer cells (Fig. 5). Since TPI1 lacks a classic NLS sequence that mediates the binding to importin transporters, how it is transported into nucleus remains an open question. It is possible that TPI1 binds to some partners that translocate between cytoplasm and nucleus thus deliver TPI1 into the nucleus. Such mechanisms have been discovered for other metabolic enzymes, such as GAPDH which interacts with Siah1 to translocate to nucleus under NO stimulation [32].

Interestingly, we found the upstream signals that promote TPI1 translocation, which are external stress conditions, such as oxidative pressure and chemotherapy drugs. In vitro and in vivo experiments demonstrated that nuclear-localized TPI1 significantly improved tumor cell resistance to chemotherapy. At least three mechanisms have been reported for metabolic enzymes to regulate gene expression. First is to regulate the local metabolites concentration in the nucleus to change histone modifications. For example, ATP-citrate lyase (ACLY) [33], acetyl-CoA synthetase 2 (ACSS2) [34] and Fumarase (FH) [35] have been reported to regulate histone modifications through their metabolic activities. Second is to act as a protein kinase and directly phosphorylate histone. For instance, the embryonic pyruvate kinase M2 (PKM2) can translocate upon EGFR stimulation, phosphorylates and promotes the acetylation of histone H3 to induce the expression of cell-cycle progression and cell proliferation genes [20]. Third is to interact with specific DNA sequence to regulate gene expression. For example, enolase 1 (ENO1) mRNA can alternatively translate a 37 kDa protein, Myc promoter-binding protein-1 (MBP-1), which binds to the c-myc P2 promoter and acts as a transcriptional repressor [36]. Our results suggested that the oncogenic activity of TPI1 is independent of its catalytic activity. In addition, bioinformatic analysis of the encoding sequence of TPI indicates TPI1 does not possess a DNA binding domain. Data from interactome database (thebiogrid.org) indicates TPI1 may interact with some nuclear proteins, such as Nanog, EZH2, PCNA, et al., suggesting it may participate in some nuclear complexes. Further study is required to elucidate the molecular mechanism how the nuclear TPI1 may promote LUAD cell growth.

The role of TPI1 in tumor development is complicated and largely unclear so far. It has been found that TPI1 is upregulated in a variety of tumors, including breast cancer, gastric cancer, and lung cancer [37, 38]. One study has reported that TPI1 could promote cell proliferation and migration in gastric cancer with mechanism unclear [24]; however, another study in liver cancer indicates that TPI1 inhibits tumor cell growth [39]. Therefore, the function of TPI1 in different tumors is complicated and inconsistent. Our work, by integrating cell observation and clinical sample analysis, demonstrate that TPI1 is important for the oncogenic capability of LUAD cells that is independent of the metabolic activity. It is of interest to explore whether similar mechanism is also applicable for TPI1 in the regulation of other types of cancers.

## Materials and methods

### Cell culture

The A549, H1299, PC9, SPAC1, and U2OS cells were purchased from the Cell Resource Center of Shanghai Institute for Biological Sciences (Chinese Academy of Sciences, Shanghai, China). The All the cells were cultured in 1640 RPMI medium (Invitrogen) containing 10% fetal bovine serum (Gibco) and 50 μg/ml of penicillin/streptomycin.

### Antibodies

Monoclonal antibody to TPI1 (sc-166785) was from Santa Cruz Biotechnology. It was used for western blot, immunohistochemistry and immunofluorescence.

### Stable cell line establish/infection

To generate A549 and H1299 stable cells with *TPI1* knockdown, shRNA oligo targeting TPI1 were custom-synthesized, annealed, and inserted into the pLKO.1 plasmid, according to standard cloning protocol. The sequences for shTPI1#1 are 5′-CCGGTCCAAACTGTATCTTCCTTTACTCGAGTAAAGGAAGATACAGTTTGGATTTTTG-3′. The sequences for shTPI1#2 are 5′-CCGGGGCCAATCCCTTCTCCACTTACTCGAGTAAGTGGAGAAGGGATTGGCCTTTTTG-3′. Lentiviruses carrying pLKO.1-vector or pLKO.1-shTPI1 was produced in HEK293T cells using psPAX2 and pMD2.G as packaging plasmids. Lentiviral supernatant was harvested from HEK293T cells 36 h after transfection and filtered with 0.45-µm filters. A549 and H1299 cells were infected with filtered lentiviral media with the addition of polybrene (8 µg/ml). Stable pools were obtained after selection with 0.5 µg/ml hygromycin.

To generate A549-shTPI1 and H1299-shTPI1 stable cells re-expressing wide-type TPI1, TPI1-NLS, TPI1-NES, or TPI1-E104D, lentiviruses carrying pCDH-vector or pCDH-TPI1 were produced and infected as described above.

### Seahorse XFe96 metabolic flux analysis

Real-time extracellular acidification rates(ECAR) rates were determined using the Seahorse Extracellular Flux (XFe96) analyzer (Seahorse Bioscience, USA). Briefly, 15000 A549 cells or 12500 H1299 cells per well were seeded into XFe96 well cell culture plates after sorting, and incubated for 12 h to allow cell attachment. After 12 h of incubation, cells were washed in pre-warmed XF assay media. Cells were then maintained in 180 μL/well of XF assay media at 37 °C, in a non-CO_2_ incubator for 1 h. During the incubation time, we loaded 20 μL of 100 mM glucose, 22 μL of 10 μM oligomycin, and 25 μL of 500 mM 2-deoxyglucose, in XF assay media into the injection ports in the XFe96 sensor cartridge. Data sets were analyzed using XFe96 software. All experiments were performed in triplicate.

### Immunofluorescence assay

Cells were washed with cold PBS and fixed with 100% Methanol for 15 min at −20 °C. Then, cells were treated with 0.2% Triton X-100 for cell perforation at room temperature for 10 min, and were incubated with blocking buffer (5% BSA in PBS) for 30 min, followed by incubation at 4 °C overnight with the primary antibody against TPI1, and Alex Fluor 488 (Green) conjugated secondary antibody (Invitrogen) at room temperature for 1 h. Cell nucleus was stained with DAPI (Invitrogen). Images were captured using Leica fluorescence optical microscope.

### Colony formation assay

A549 and H1299 cells were seeded in 12-well plates (250 cells/well and 300 cells/well, respectively). The plates were placed in the incubator and the medium was changed every 2 days for 10 days. Then the colonies were fixed by 4% paraformaldehyde in PBS and stained with 0.05% crystal violet solution.

### Cell migration assay

Transwell chambers with 8-µm pores for 24-well plates were used in this assay. Cells were suspended in serum-free DMEM and seeded into chambers at 30,000 cells per well. The bottom chamber was filled with 1640 medium supplemented with 10% FBS. After seeding for 24 h, the membranes were fixed in 4% paraformaldehyde and stained with 0.05% crystal violet. Photos were taken by using an Olympus microscope and migrated cells were counted and quantified.

### Immunoblotting

Cells were lysed in SDS sample buffer and denatured by heating on 100 °C for 10 min. Proteins were separated on 8% to 10% Bis-Tris polyacrylamide gels. Western blot images were captured by typhoon FLA 9500 (GE Healthcare).

### Tissue IHC

Paraffin-embedded lung cancer and matched normal lung tissue microarrays were obtained from Shanghai Zhongshan Hospital, Fudan University. Paraffin-embedded slides were deparaffinized in xylene for two times, 3 min each, rehydrated in a series of ethanol solutions. After two washes in PBS for 5 min each, antigen retrieval was performed in Citrate Antigen Retrieval Solution (Beyotime) by boiling for 10 min. After cooling down to RT and rinsing with PBS for two times, the slides were blocked with 10% fetal bovine serum (FBS) in PBS for 1 h at RT. Then, various primary antibodies were applied in a concentration of 8 μg/ml overnight at 4 °C. After wash with PBS, horseradish peroxidase (HRP)-conjugated secondary antibodies were added onto the slides for 1 h at RT. DAB substrate solution was used to reveal the color of antibody staining.

### Xenograft injection

For Xenograft experiments, nude mice (BALB/c, female, 5 weeks old; Shanghai SLAC Laboratory Animal) were subcutaneously injected with 1 × 10^7^ of control, TPI1 knockdown, TPI1-NLS, or TPI1-NES cells. Mice were sacrificed 2 weeks after A549 cells injection or 3 weeks after H1299 injection, respectively. The xenograft tumors were dissected and analyzed.

Animals were randomly assigned to subcutaneous injection. Xenograft tumor experiments on mice were approved by the Animal Care and Use Committee at Fudan University.

### Quantification and statistical analysis

Statistical analyses were performed using one-way analysis of variance (ANOVA) with Bonferroni’s test or Student’s *t*-test. All data shown represent the results obtained from triplicated independent experiments with standard errors of the mean (mean ± SD). The values of *p* < 0.05 were considered statistically significant.

### Human study approval

Written informed consent was obtained from each LUAD patients who underwent surgery at the Department of Thoracic Surgery, Zhongshan Hospital, Fudan University, Shanghai, China. This study was approved by the Zhongshan Hospital Research Ethics Committee (No. B2021-131).

## Supplementary information

Reproducibility checklist

Supplementary Figures

Original gel Data for Western Blot data

## Data Availability

All data needed to evaluate the conclusions in the paper are present in the paper. Additional data related to this paper may be requested from the corresponding author.
