# Supplementary material for: Elevated nuclear localization of glycolytic enzyme TPI1 promotes lung adenocarcinoma and enhances chemoresistance
Source: Cell Death Dis. 2022 Mar 4;13(3):205. doi: 10.1038/s41419-022-04655-6 (PMC8897412; doi:10.1038/s41419-022-04655-6)
Supplement: Supplementary file 2 — Supplementary Figures [file 41419_2022_4655_MOESM2_ESM.pdf]

Supplementary Table 1

Patient ID	Gender	Age(years old)	Clinical Stage
1	Female	66	Ia
2	Male	52	Ib
3	Male	58	Ib
4	Male	59	IIb
5	Male	69	Ib
6	Female	58	Ib
7	Male	80	IV
8	Female	66	Ib
9	Male	81	Ia
10	Male	70	Ia
11	Female	49	IIIa
12	Female	62	Ia

Table S1. Clinical information of LUAD patients.

Supplementary Figure 1

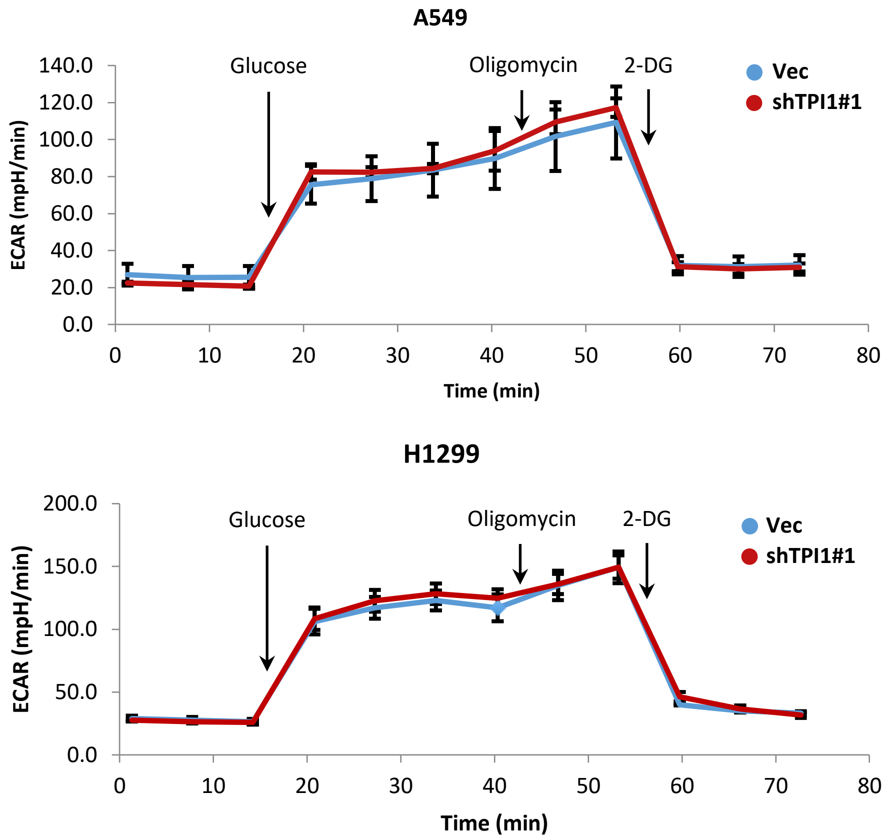

Figure S1. Loss of TPI1 does not affect glycolysis. ECAR was measured by Seahorse XF assays in both A549 and H1299 cells after treated with shTPI1#1 or scramble shRNA.

Supplementary Figure 2

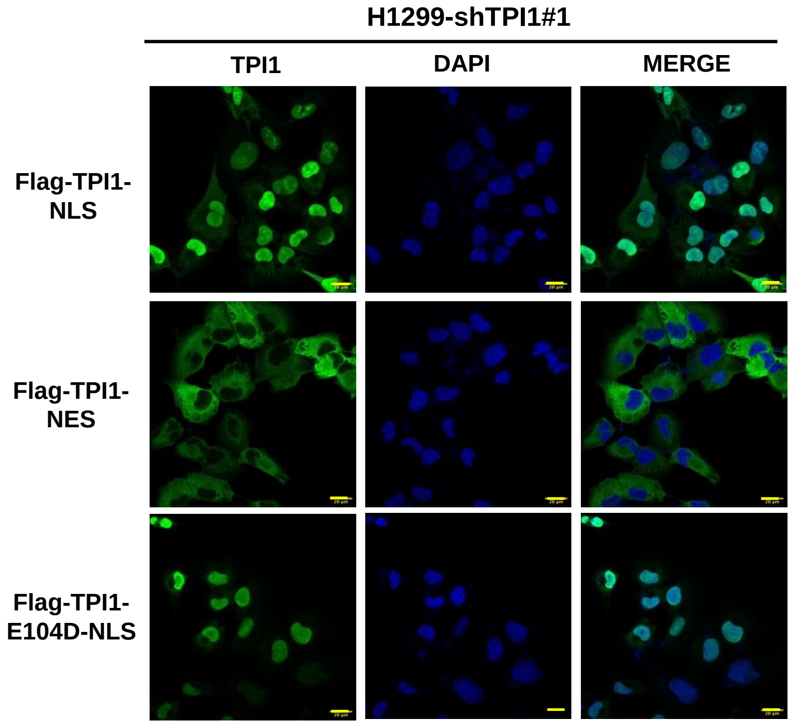

Figure S2. Validation of localization of TPI1-NLS, TPI1-NES and TPI1-E104D-NLS in H1299 cells by immunofluorescence staining. Scale Bars: 50 μm.

Supplementary Figure 3

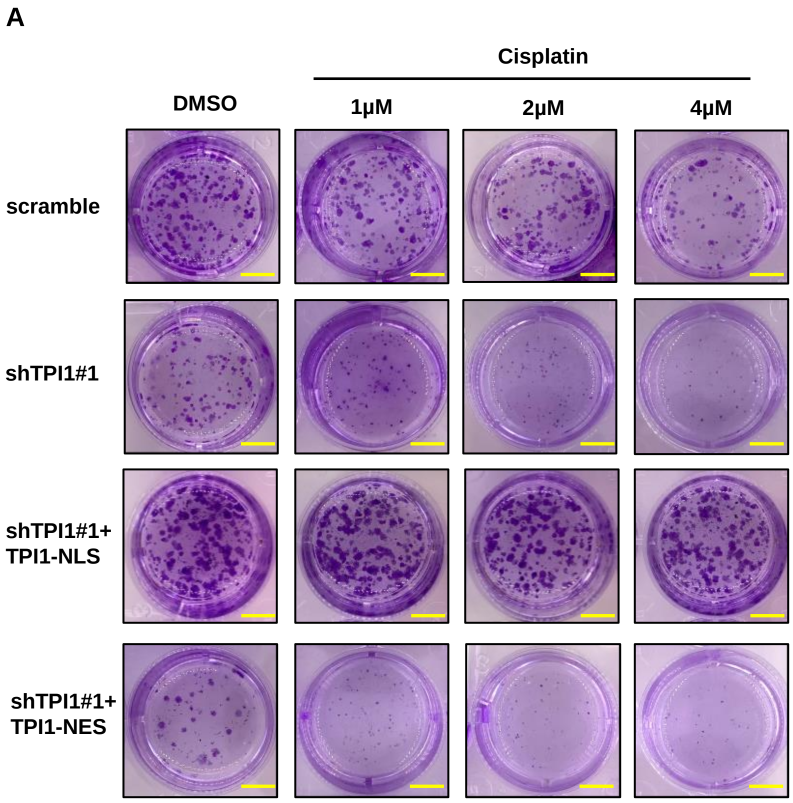

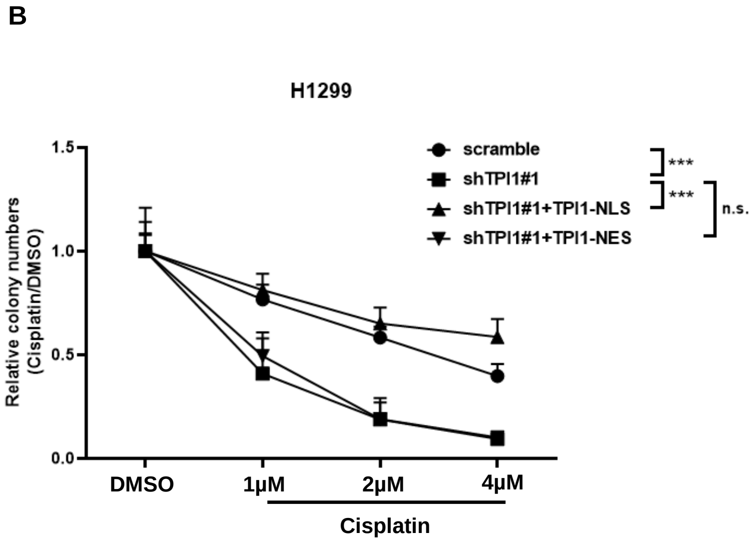

Figure S3. TPI1-NLS, but not TPI1-NES, rescued cells from ultra-sensitivity to cisplatin caused by TPI1 knocking down in H1299 cell. (A) Colony formation upon cisplatin treatment in Scramble, TPI1 knockdown, TPI1-NLS or TPI1-NES expressing cells. (B) Quantification of colony formation results in (A). Asterisks denote statistical significance with one-way ANOVA. $^{**}p < 0.01$; $^{***}p < 0.001$ for the indicated comparison.
