# Supplementary figures and images for: Elevated nuclear localization of glycolytic enzyme TPI1 promotes lung adenocarcinoma and enhances chemoresistance
Source: Cell Death Dis. 2022 Mar 4;13(3):205. doi: 10.1038/s41419-022-04655-6 (PMC8897412; doi:10.1038/s41419-022-04655-6)

Figure 1F

TPI1

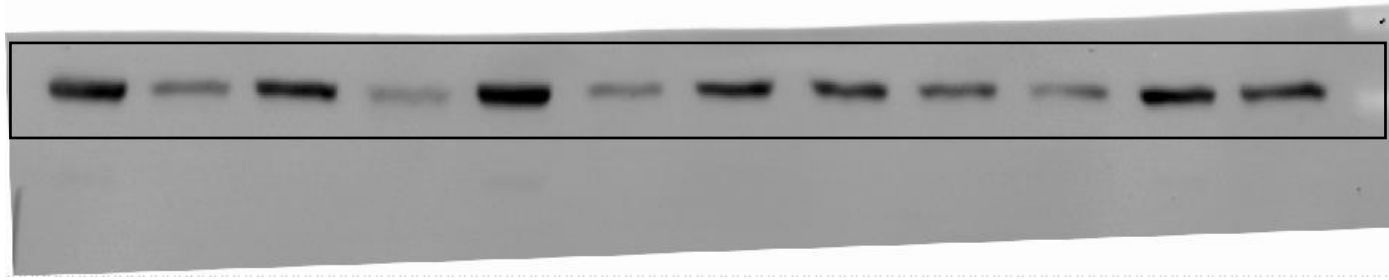

ACTIN

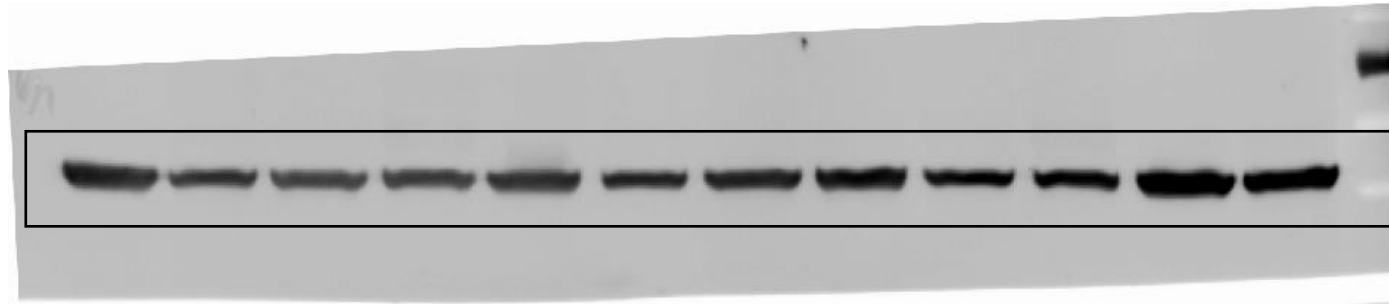

TPI1

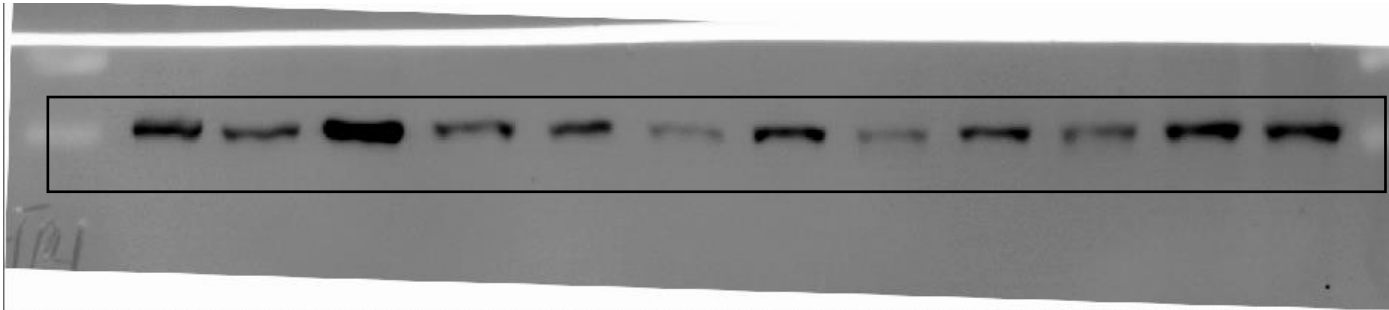

ACTIN

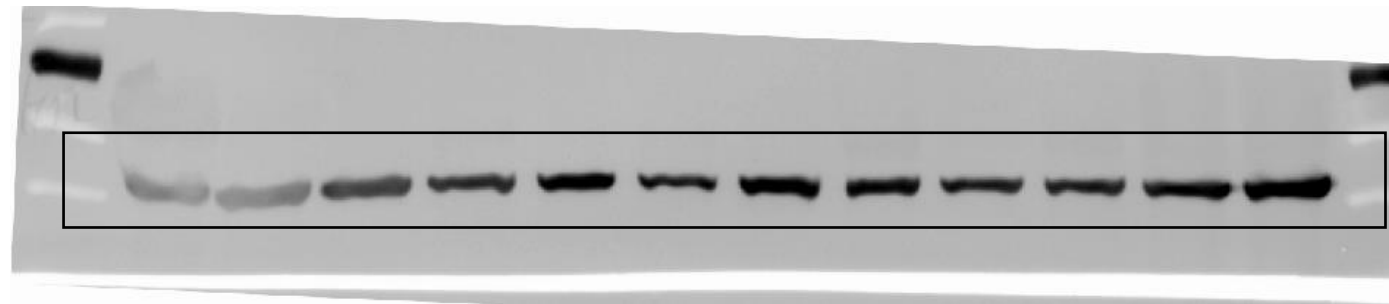

Figure 2A

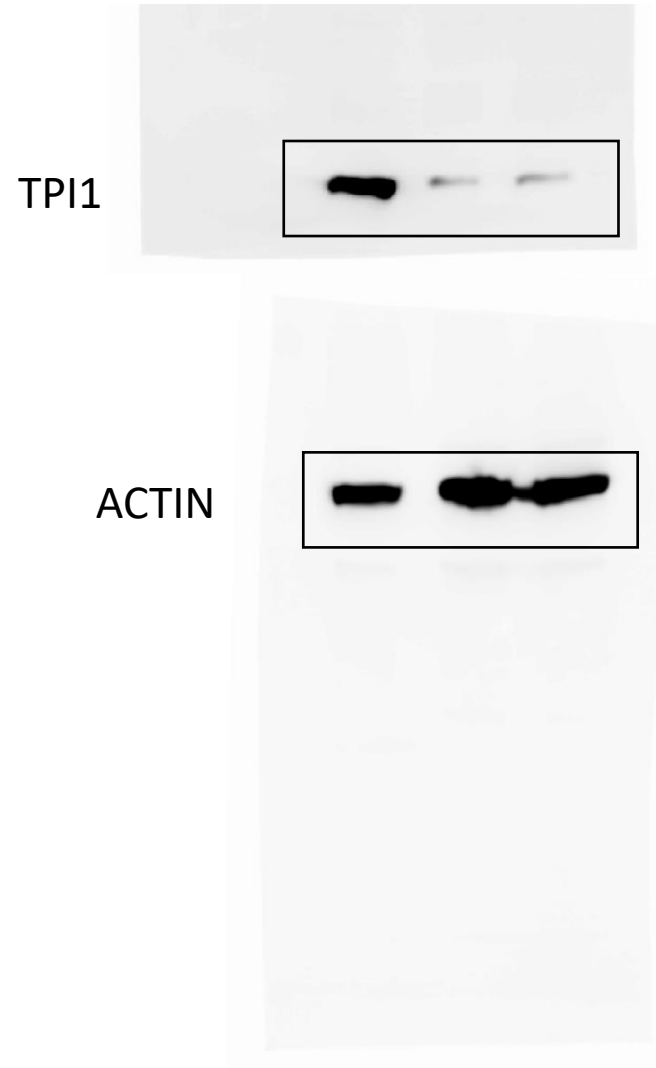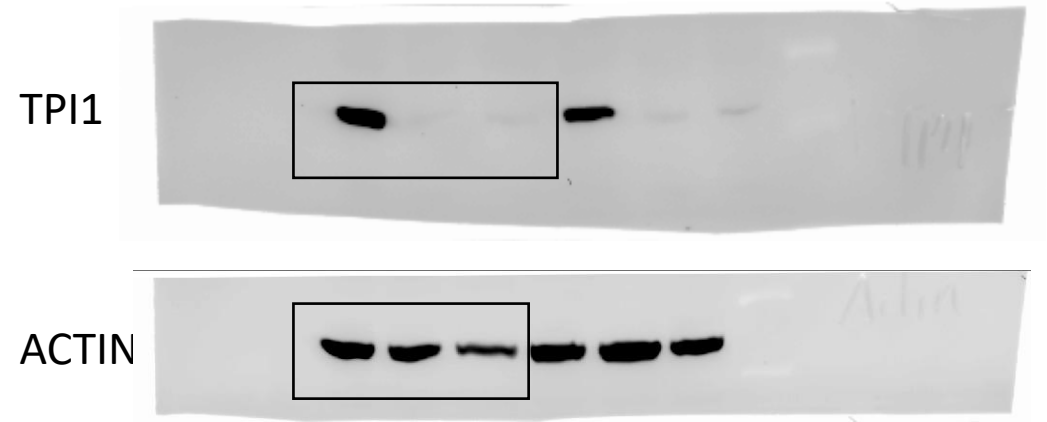

Figure 3A

TPI1

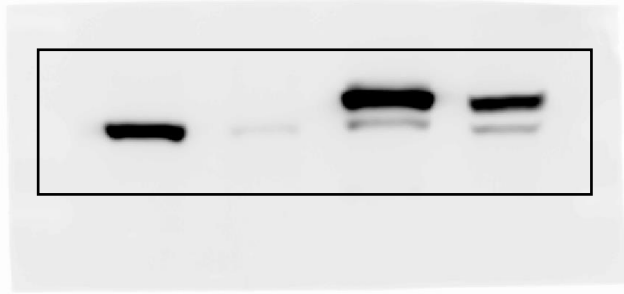

TPI1

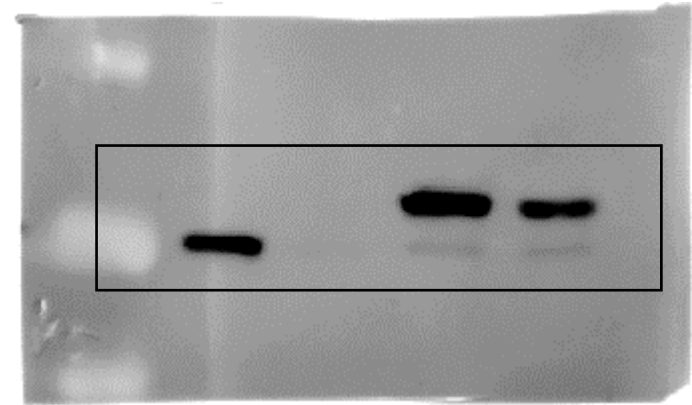

ACTIN

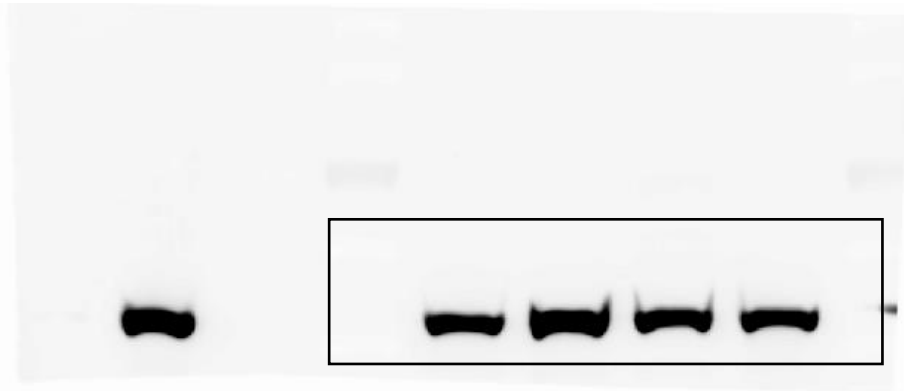

ACTIN

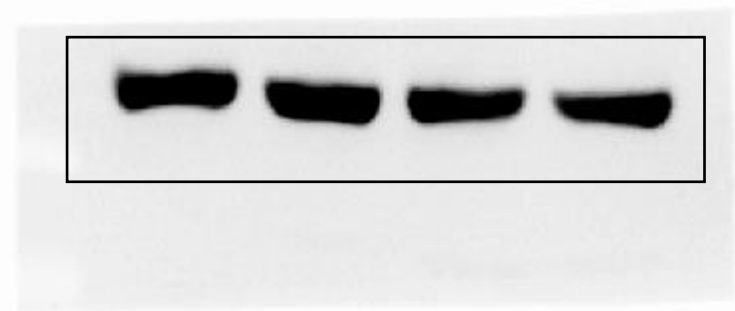

Figure 5A

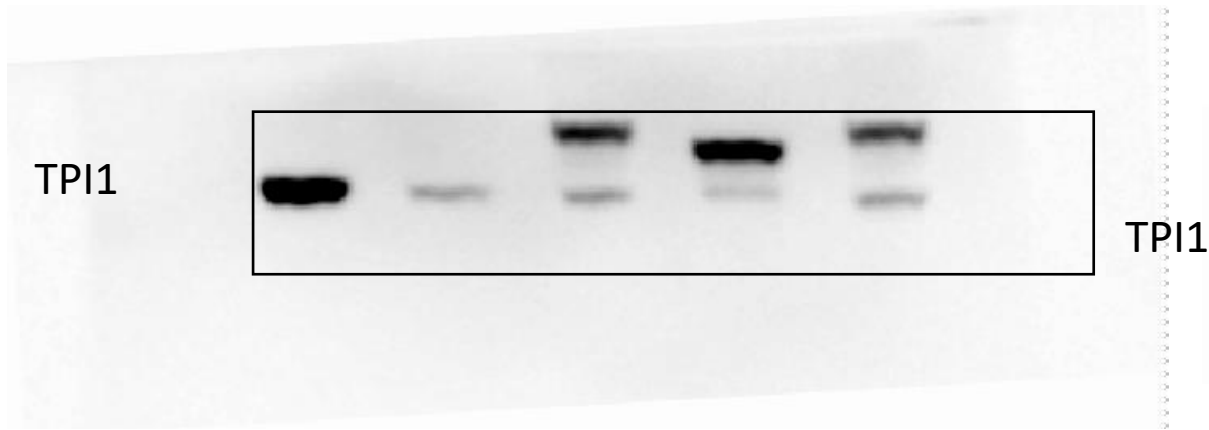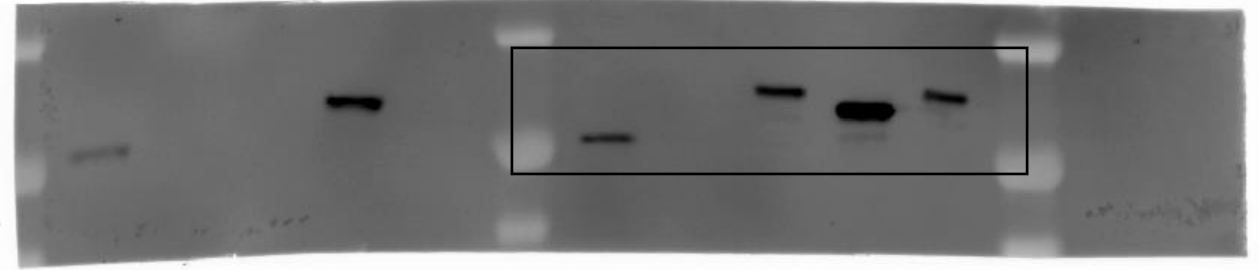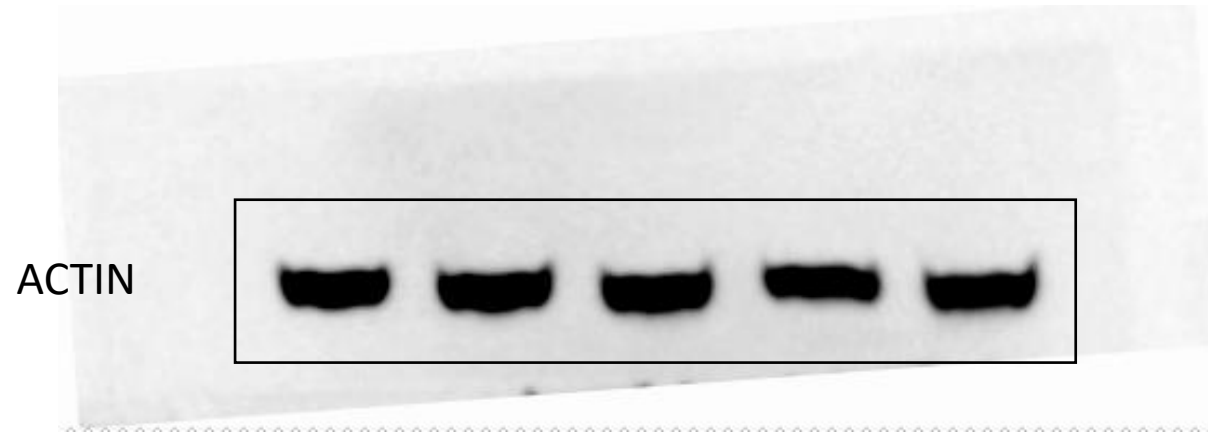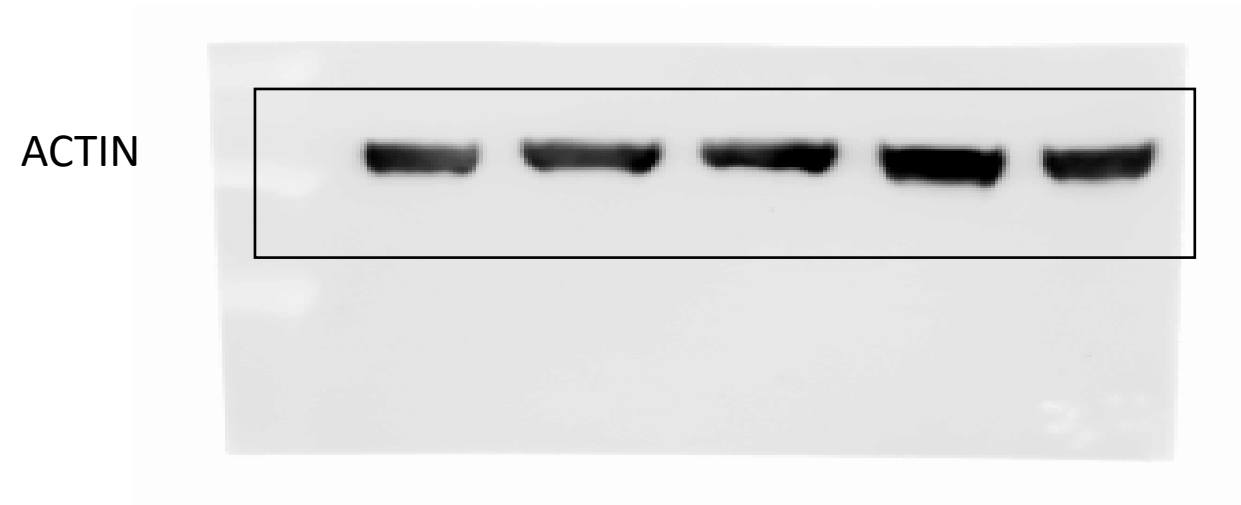

Supplement: Supplementary file 3 — Original gel Data for Western Blot data [file 41419_2022_4655_MOESM3_ESM.pdf]
